# Mass spectrometry coupled to imaging techniques: the better the view the greater the challenge

**DOI:** 10.3389/fphys.2015.00003

**Published:** 2015-01-22

**Authors:** Gwendolyn Barceló-Coblijn, José A. Fernández

**Affiliations:** ^1^Lipids in Human Pathology, Research Unit, Hospital Universitari Son Espases, Institut d'Investigació Sanitària de Palma (IdISPa)Palma, Spain; ^2^Departamento de Química-Física, Facultad de Ciencia y Tecnología, Universidad del País Vasco (UPV/EHU)Leioa, Spain

**Keywords:** mass spectrometry (MS), Lipid imaging, MALDI Imaging, SIMS, DESI imaging

## Abstract

These are definitively exciting times for membrane lipid researchers. Once considered just as the cell membrane building blocks, the important role these lipids play is steadily being acknowledged. The improvement occurred in mass spectrometry techniques (MS) allows the establishment of the precise lipid composition of biological extracts. However, to fully understand the biological function of each individual lipid species, we need to know its spatial distribution and dynamics. In the past 10 years, the field has experienced a profound revolution thanks to the development of MS-based techniques allowing lipid imaging (MSI). Images reveal and verify what many lipid researchers had already shown by different means, but none as convincing as an image: each cell type presents a specific lipid composition, which is highly sensitive to its physiological and pathological state. While these techniques will help to place membrane lipids in the position they deserve, they also open the black box containing all the unknown regulatory mechanisms accounting for such tailored lipid composition. Thus, these results urges to different disciplines to redefine their paradigm of study by including the complexity revealed by the MSI techniques.

## Introduction

Lipids are a very heterogeneous family of molecules that were initially classified as those compounds not soluble in water but soluble in organic solvents. At first glance, they are structurally very different: cholesterol, sphingolipids, phospholipids, triacylglycerides are just some of the subfamilies all classified under the name of lipids. At the same time, lipid extracts are complex mixtures to analyze because of the close structural relationship existing among many of them. Thus, lipid species may differ only by the position of a single double [i.e., alpha-linolenic acid (18:3n-3) vs. gamma-linolenic acid (18:3n-6)]. These simple differences may involve totally different biosynthetic pathways, functions and tissue (even cell) location. This complexity is one of the reasons, although not the only one, why lipid research has fallen behind compared to protein and gene research (Muro et al., 2014). In this sense, lipid analytical techniques have improved considerably in the last decades. However, a technique similar to the existing for proteins allowing lipid visualization was still missing. It is clear that to elicit the spatial distribution of the molecular entities and their dynamics during the developmental cycle is crucial to understand the biochemical complexity occurring in living organisms (Cornett et al., 2007). There are many examples involving proteins. Thus, an increased translocation of beta-catenin from cytoplasm to nucleus is associated with the malignant transformation of the cell, despite that the total beta-catenin may have not changed. Surprisingly, to convince that an equivalent situation is happening with lipids species is not being an easy task. The description of some lipid microdomains, as lipid rafts, has introduced some order but, even so membrane lipids distribution within cell membranes is still perceived as a non-regulated event.

Lipid visualization using techniques based on fluorescent or confocal microscopy is possible. Thus, different chemical compounds as antibodies (for ceramides, Vielhaber et al., 2001 glucosylceramides, Toledo et al., 2001 phosphatidyl inositides, Thomas et al., 1999 and oxidized lipids detection Palinski et al., 1996), proteins (for sphingomyelins detection, Shogomori and Kobayashi, 2008) and polyene macrolides (for cholesterol detection, Schroeder et al., 1971) have been described. However, their use is limited as they detect lipid groups rather than single molecular species. Additionally, the use of external probes constrains the detection to known substances and it perturbs in some degree the existing lipid-lipid and lipid-protein interactions. In this case, lipid researchers seem to have been lucky because the combination of soft-ionization MS and imaging techniques (MSI) turned out particularly suitable for detailed lipid analysis (Cornett et al., 2007; Jackson et al., 2007a,b).

**Figure 1:**
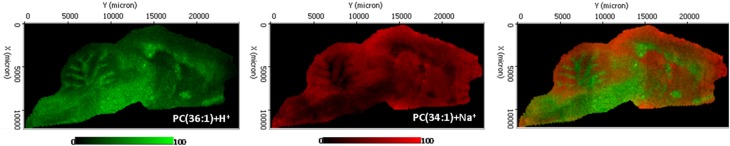
**Distribution of two phosphatidylcholine molecular species in sagittal section of rat encephalon, recorded in positive ion mode using an Orbitrap analyzer**. The section was ~25 × 10 mm and the spectra were recorded with 100 μm spacing, resulting in 28,000 spectra. Ten shots were averaged for each pixel. Mass resolution was 60,000.

Hence, in addition to provide the precise lipid composition, MSI allows mapping each detected analyte within a tissue section. MSI would be the equivalent to immunochemistry or fluorescent microscopy techniques for the study of the spatial arrangement of molecules with the great advantage that no probes or labels are necessary (Stoeckli et al., 2001). However, the beautiful images obtained by MSI bring to light the great deficiencies existing in the lipid field regarding the knowledge about the function that each individual lipid species may have and about the regulatory mechanisms controlling the specific distribution (Figure 1). The good news is that the advances in different fields including not only MSI analysis, but also microscopy and biophysics, allows us to be optimistic and it may foresee a new scenario in which lipid researchers will start to untangled the mechanisms underlying the lipid diversity (Muro et al., 2014).

## Lipid analysis evolution

Curiously enough, lipids were one of the first groups of biomolecules to be studied and analyzed back to the XVII and XVIII centuries. However, the interest for them stopped abruptly after DNA and gene code discovery. In addition to the great relevance of the latter events, lipids have been always a tough family to analyze. Lipid extracts contain hundreds of different but highly related molecules that may differ just in the presence or absence of a single double bound, which means a difference of ±2 Da in molecular weight. First lipid analyses were done by thin layer chromatography (TLC). By using different solvents systems the separation of major lipid classes, phospholipids, sphingolipids, and neutral lipids can be achieved. However, each TLC band contains numerous molecular species. Later, Gas Chromatography (GC) and High Performance Liquid Chromatography (HPLC) were developed but the low volatility of lipids and the lack of a fluorophore moiety prevented the use of these methodologies for direct lipid analysis as derivatization methods were needed, some of them quite tedious and time-consuming (Brockerhoff, 1975; Fine and Sprecher, 1982; Takamura and Kito, 1991). Direct analysis has been only possible once the use of MS became more frequent.

Next, the three most common desorption ionization MS techniques applied to lipid analysis, Secondary Ion Mass Spectrometry (SIMS), Matrix Assisted Laser Desorption Ionization (MALDI) and Desorption Electrospray Ionization (DESI) will be described, including briefly their advantages and disadvantages. Interestingly for lipid researchers the three methodologies resulted particularly suited for lipid analysis (Passarelli and Winograd, 2011). For further detailed information many quite extensive and thorough reviews of each technique are available (McDonnell and Heeren, 2007; Amstalden van Hove et al., 2010; Pol et al., 2010; Vickerman, 2011). The continuous effort of the MS community to improve the sensitivity of the methods has given rise to a number of emerging and very promising desorption/ionization techniques (Harris et al., 2011) such as rapid evaporative ionization MS (REIMS) (Schafer et al., 2009) and laser ablation electrospray ionization (LAESI) (Shrestha et al., 2010).

## Desorption ionization MS and lipids

First lipid analysis using desorption ionization MS techniques date back to the 60's and 70's (Ryhage and Stenhagen, 1960; Lauer et al., 1970). SIMS was developed (Benninghoven, 1967) in the late 60's and nowadays is a powerful technique for molecular surface analysis which has been successfully applied to tissue sections. SIMS uses a beam of high-energy ions (primary ions) to bombard the surface of the sample which induces the sputtering of molecules and the ionization of small proportion of the analytes (<0.1%, secondary ions) which are the ones detected (Vickerman, 2011). SIMS uses a focused ion beam of individual or clusters of high-energy particles, such as Bi^+^, Au^3+^, C^+^_60_, CF^+^_3_, SF^0^_6_, or SF^+^_5_ (Vickerman, 2011; Watrous et al., 2011). The high sensitivity attributed to SIMS allows for the detection of lipid molecules at attomolar (10^−18^) concentrations (Braun et al., 1999). However, SIMS has yet to be readily applied to the analysis of biofluids and tissues because of the existence of analyte fragmentation generated by the high-energy primary ion beam and its limited sensitivity at >500 Da (McDonnell and Heeren, 2007). This problem was partially overcome thanks to development of soft ionization techniques, such as MALDI (Karas and Hillenkamp, 1988; Tanaka et al., 1988) and DESI mass spectrometry (Takats et al., 2004, 2005). While spatial resolution is lower compared to SIMS (see below), these techniques induce less molecular fragmentation, allowing the detection of the m/z of whole lipid molecules. Importantly, in all three methods, the final peak assignment requires accurate mass measurement and tandem MS/MS methods to be applied. In any case, important advances have occurred in the last years in both SIMS methodology and instrumental allowing this technique to provide critical information regarding biological samples, in particular regarding single cell composition (Vickerman, 2011).

In MALDI-MS, the analyte is co-deposited with a low-molecular-weight organic matrix, which absorbs the laser radiation, assisting in the emission of molecular ions and fragment ions (Karas and Hillenkamp, 1988; Tanaka et al., 1988). Limits of detection in MALDI-MS may be as low as 10^−15^–10^−21^ mole, which makes this technique ideal for the measurement of compounds in biological samples (Rubakhin et al., 2005). There may be concerns regarding the stability of some lipid species during the analysis, because most of the analyses are done in the absence of crioprotective substances. The fact is that the vacuum environment existing inside the MS should prevent lipid oxidation while the acidic environment provided by the aqueous solution containing the matrix inhibits the enzymatic activity that could affect the lipid composition. In addition to absorb the energy from the laser beam and transfer it into excitation energy, the matrix also isolates the analyte molecule from one another, reducing the analyte aggregation to minimal level (Fuchs et al., 2010). The most critical step during MALDI measurements is the sample preparation, because an uneven distribution of the matrix may lead to experimental artifacts. However, matrix sublimation can circumvent this problem (Hankin et al., 2007). The heterogeneous matrix crystallization and laser energy fluctuation results in a poor ion-signal reproducibility which makes very difficult to obtain quantitave results using MALDI (Pirman et al., 2013). In the lipid analytical field, the most widely used matrix is 2,5-dihydroxybenzoic acid (DHB, Jackson et al., 2005; Hankin et al., 2007) although different alternatives providing better results have been described as α-cyano-4-hydroxycinnamic acid (αCHCA), 2-mercaptobenzothiazole (MBT, Astigarraga et al., 2008) and 2,5-diaminonaphtalene (DAN, Thomas et al., 2012). Finally, the vacuum conditions needed for the analysis may constrain to some degree the use of this technique. Taking this into account, a new set of techniques has emerged, in which mass spectra are obtained under ambient conditions and the usage of a matrix to mediate desorption and ionization is avoided (Luo et al., 2013). In this regard, Desorption Electrospray Ionization (DESI) is currently the most extended technique (Gerbig et al., 2012; Eberlin et al., 2013).

DESI is based on the electrospray ionization (ESI) methodology develop by Fenn et al. (1989). In this arrangement, the charged droplets and ions produced in an electrospray jet are directed at the surface of the analyte to desorb the ions (Takats et al., 2004). Although the mass spectrometer and its detector still require of a vacuum system for operation, desorption and ion formation processes can take place at atmospheric pressure, with full access to the sample during acquisition (Takats et al., 2004; Cooks et al., 2006). In addition to a lower sensitivity, the main limitation of DESI is spatial resolution (see below) because “focusing” the charged solvent spray is difficult (Wiseman et al., 2008). In any case, their introduction was a significant step toward the direct, *in situ* mass spectrometric investigation of delicate samples including living biological systems (Gerbig et al., 2012). DESI has been shown to be effective for the detection of a wide range of lipid classes (Manicke et al., 2008; Dill et al., 2009; Wu et al., 2009) making it ideal for lipid-imaging applications (Blanksby and Mitchell, 2010).

Independently of the method of ionization chosen, the large amount of molecules present in tissues exceeds usually the ability of the spectrometers to separate them, unless an FTICR (Fourier transform ion cyclotron resonance) analyzer with a state-of-the-art magnet is used. However, it is possible to include a post-ionization selection, by introducing an ion mobility cell, which allows discriminating between isobaric molecules with different collision cross sections (Jackson et al., 2014).

## Desorption ionization MS coupled to imaging techniques: MSI

Currently, all SIMS-, MALDI-, and DESI-MS techniques are adapted to imaging. In MSI, basically, mass spectra are acquired by stepping the microprobe (focused laser beams, ions currents…) across the sample, typically in a raster pattern, to read the details of its chemical composition, in our case the complete lipidome. Then, using different software, images of the distribution of a given analyte [characterized by mass-to-charge (*m/z*) values] can be generated in which each pixel is colored using a scaled false color according to its relative intensity (Rubakhin et al., 2005; Watrous et al., 2011). Importantly, a high correlation between the resulting molecular images and the histology of the tissue sections is maintained (Thomas et al., 2013).

The primary advantage of SIMS is its high spatial resolution (as small as 50 nm), a powerful characteristic for tissue imaging with MS (Boxer et al., 2009). In MALDI- and DESI-MSI spatial resolution is lower. Various efforts have been made to improve the spatial resolution of MALDI imaging, including oversampling (Jurchen et al., 2005), laser modulation (Holle et al., 2006) (i.e., smart beam technology) and solvent-free sublimation matrix application (Hankin et al., 2007). Despite these efforts, neither MALDI nor DESI has not achieved the spatial resolution of SIMS yet, and typically work on the 10–100 μm and ~ 200 μm regime respectively (Ifa et al., 2007; Murphy et al., 2009; Deeley et al., 2010; Kettling et al., 2014). Conversely, in terms of chemical specificity, MALDI and DESI techniques cover a broader range of biomolecules—including proteins, peptides, lipids, and nucleotides. Altogether, MSI is a versatile analytical tool that enables multiplexed, non-targeted, and label free molecular imaging of biological specimens (Lanni et al., 2014).

It is clear that MSI provides a powerful technique to start to understand basic membrane lipid physiology, going beyond the barrier function or the effect on membrane fluidity. It has been shown for many years that different tissues present different lipid composition, especially regarding main lipid classes. However, it was hard to imagine the great level of specificity and accuracy that MSI images are revealing. Thus, beautiful images showing the absolute specific distribution of certain lipid molecular species within the brain and spinal cord (Landgraf et al., 2009; Koizumi et al., 2010) have been obtained. Consistently, this specificity applies for any type of lipid: polyunsaturated fatty acids (Sugiura et al., 2009), phospholipids (Veloso et al., 2011a,b) and sialylated gangliosides and glycosphingolipids (Sugiura et al., 2008; Chan et al., 2009). Still, little can be said about the physiological reasons of this distribution, as we do not know the real function of each individual molecular species. In fact, there is so little knowledge in the role of membrane lipids in physiology that any tissue is worth being studied: human lens and retina (Ellis et al., 2010; Pol et al., 2011; Zemski Berry et al., 2014), lungs (Berry et al., 2011), adrenal glands (Wu et al., 2010), bladder tissue (Dill et al., 2009), colon (Brulet et al., 2010). The complex and specific composition revealed by all these studies evidence the great level of regulation in regards of lipid synthesis, transport and degradation.

Furthermore, MSI offers a powerful tool to understand the role that lipids have in pathological alterations such as cancer (Dill et al., 2009; Eberlin et al., 2013; Ide et al., 2013; Kurabe et al., 2013; Thomas et al., 2013; Waki et al., 2014), neurodegenerative and mental diseases (Matsumoto et al., 2011), tissue damage after ischemic insults(Koizumi et al., 2010; Hankin et al., 2011), myocardial infarction (Menger et al., 2012), microbial infection (Qureshi et al., 2010), colon (Brulet et al., 2010; Gerbig et al., 2012; Kurabe et al., 2013). Thus, MSI analysis of neoplastic lesions allow visualizing what it was already demonstrated using other techniques lacking spatial resolution: tumor lipid composition differs of non-tumor tissues and consequently tumor boundaries could be easily established (Ide et al., 2013; Kurabe et al., 2013). The interesting challenge here is to find out if it is possible to establish a tissue independent lipid profile associated to the malignant transformation occurring during a tumor formation.

Finally, the common most questions for MSI experts: is it possible to analyzed single cells? The answer is yes, but only using SIMS meaning that, the available data represents the distribution of molecular fragments separately as fatty acids, lipid head groups… (Gerbig et al., 2012; Eberlin et al., 2013; Lanni et al., 2014; Waki et al., 2014). Certainly, despite that SIMS allows achieving resolutions as low as 50 nm, this occurs at the expenses of using mono atomic beams that result in extensive fragmentation. As it happens with MALDI, the settings in a SIMS-MSI experiment are a compromise between spatial resolution and identification of biomolecules: polyatomic beams allow one to detect and identify intact metabolites but at a lower resolution (still at hundreds of nm) (Cheng et al., 2007; McDonnell and Heeren, 2007).

The great translational applicability that MSI techniques may have is clear (Watrous et al., 2011). Currently, clinical methods based on imaging, histology, autoradiography of radiolabeled compounds and fluorescence microscopy need labels, have lower resolution, specificity and limited number of target compounds can be monitored at once (Cornett et al., 2007). MSI has the potential to complement these methods and great efforts are now being made to develop the required technology to bring the MSI into the operational rooms (Eberlin et al., 2013).

The pitfalls, advantages and successful applications of each MS technique have been already briefly mentioned. Regarding MSI techniques the main and common limitation is that they cannot be applied in living biological systems because they are invasive and samples are consumed during the analysis. However, the quantity and quality of the provided output may compensate this deficiency. MSI techniques also share common challenges: to increase in sensitivity (in most of the cases only major lipid classes are detected), to increase of the spatial resolution, to improve the available software to handle the large and complex amount of data and to establish a clear normalization and analytical criteria and to obtain quantitative data. Many groups are actively working in establishing a reliable protocol to quantify metabolites directly from the tissue sections, but so far, any quantification data must be backed up by data obtained with more reliable sources, such as LC/MS on extracts from the same type of samples. A major concern that may arise within the lipid field is how comparable are the results obtained using different techniques. In fact, the three techniques offer different aspects of the composition of a given sample. SIMS yields the highest spatial resolution but allows for identification of a lower number of species. On the other hand, DESI does not allow achieving high resolutions, but as it involves extraction it probably allows for detection of the largest number of species. Thus, MALDI-MSI is probably mid-way, giving the identification of a large number of species at a reasonable spatial resolution. When comparing published results, it is important to keep in mind the differences in resolution of each technique (1–2 orders of magnitude). Thus, while each single pixel in an SIMS image represent the distribution of an analyte in a small part of a single cell, in MALDI may represent the average composition of 2, even one cell (manuscript in preparation) and finally in DESI may represent the average of 4 to 6 cells. Finally, it is clear that 2D images of a tissue section are a just a snap shot of what is occurring in biological samples. Big efforts are being made to obtain not only 3D MSI images (Trede et al., 2012; Norris and Caprioli, 2013) but also to combine different imaging modalities, as fluorescent microscopy or histological staining, by using multimodal molecular imaging to provide more detailed anatomical information (Chughtai et al., 2012; Thiele et al., 2014).

## Conclusions

Thanks to MSI we are finally able to establish and visualize the lipid composition of tissue structures even of single cells. It is clear that the MSI data obtained so far supports what lipid researchers have stated for years: lipid composition is far of being a random event. In fact, the exact lipid composition of a cell would be equivalent to its ID as it is unique and totally cell type-dependent. This necessarily has to do with the specific function of each type of cell within a tissue. Unfortunately, our knowledge on the role of each particular lipid molecular species is still very limited and it will take some time to really understand the functional reasons of the high specificity displayed by lipid distribution. Furthermore, it is clear that this composition is highly sensitive to the cell pathophysiological state and consequently it will vary depending if cells are undergoing cell division, differentiation, or any malignant transformation. Therefore, MSI provides us new and critical set of elements to address the studies on the mechanism underlying these processes, elements that have rarely been taken into account: membrane lipids.

Finally, these data urge to the different disciplines dedicated to lipid studies, biochemistry, biophysics, molecular biology, genetics, etc. to rethink their strategy taking into account the complex scenario revealed by MSI. Certainly, the exquisite specificity in lipid distribution can be only achieved through a complex set of enzymes that synthesizes and distributes each single lipid molecular species in a highly regulated manner. However, there are still many important questions that remains unsolved and that need to be addressed in order to understand what we are seeing: which are these enzymes? How is their activity regulated? How is their expression regulated? Fortunately there have been advances in many other techniques that will be extremely useful to improve our knowledge in lipid biology (Muro et al., 2014).

### Conflict of interest statement

The authors declare that the research was conducted in the absence of any commercial or financial relationships that could be construed as a potential conflict of interest.
